# Occurrence and antimicrobial susceptibility patterns of *Salmonella* species from poultry farms in Ibadan, Nigeria

**DOI:** 10.4102/ajlm.v11i1.1606

**Published:** 2022-07-20

**Authors:** Terese G. Orum, Olayinka O. Ishola, Oluwawemimo O. Adebowale

**Affiliations:** 1 Department of Veterinary Public Health and Preventive Medicine, Faculty of Veterinary Medicine, University of Ibadan, Ibadan, Nigeria; 2 Department of Veterinary Public Health and Preventive Medicine, College of Veterinary Medicine, Federal University of Agriculture, Abeokuta, Ogun State, Nigeria

**Keywords:** *Salmonella* species, antibiotics, antimicrobials, resistance, poultry and infection

## Abstract

**Background:**

*Salmonella* species are among the major foodborne pathogens causing diseases of economic and public health implications in poultry and humans globally.

**Objective:**

This study aimed to determine the occurrence and antimicrobial susceptibility patterns of *Salmonella* isolates from chickens in poultry farms in Ibadan, Southwest Nigeria.

**Methods:**

Cloacal swab samples (*n* = 360) were obtained from chickens randomly selected from 10 poultry farms in five local government areas of Ibadan, Oyo State, from 04 April 2018 to 20 November 2018. Bacterial identification and antimicrobial susceptibility testing were performed using established protocols. Data were analysed using descriptive statistics and Pearson’s chi-squared test at *P* ≤ 0.05 significance level.

**Results:**

The overall prevalence of *Salmonella* was 21.4%. There were statistically significant associations between *Salmonella* prevalence and the farm location (*p* = 0.003), age of chickens (*p* < 0.001), and health status of chickens (*p* < 0.001). All *Salmonella* isolates (*n* = 77; 100.0%) were resistant to cefuroxime. The isolates were also highly resistant to cotrimoxazole (*n* = 74; 96.1%), chloramphenicol (*n* = 73; 94.8%), meropenem (*n* = 72; 93.5%), gentamicin (*n* = 69; 89.6%), and tetracycline (*n* = 64; 83.1%).

**Conclusion:**

The presence of drug-resistant *Salmonella* in commercial layer chickens in Ibadan is a potential threat to consumer health as it increases the risk of carcass contamination and pathogen propagation, and limits the options to control and treat infections in humans and animals. Well-integrated national surveillance systems for monitoring *Salmonella* and antimicrobial resistance in poultry are critical.

## Introduction

*Salmonella* species is one of the primary causes of foodborne illnesses in humans globally.^1^ Although most cases of salmonellosis are mild, some may lead to life-threatening enteric fever or an invasive form of the disease requiring antimicrobial treatment, especially among immunocompromised people, infants, and the elderly.^2,3^ The consumption of food animal products such as poultry meat has been implicated in human salmonellosis outbreaks worldwide.^4^

The *Salmonella* genus comprises two species, *Salmonella bongori* and *Salmonella enterica*, and over 2500 different serotypes or serovars.^5^ In Africa, non-typhoidal salmonellae are common causes of bacterial bloodstream infections in humans with clinical signs of fever^6^ and are associated with case fatality rates of about 20.0% – 25.0%.^7^ A report from Nigeria showed that non-typhoidal salmonellae (43.5%) and typhoidal salmonellae (56.5%) were isolated from blood and stool samples collected from adults and children.^8^

Poultry farming is a major source of livelihood for poor rural households and urban areas in many developing countries, including Nigeria.^8,9^ The poultry sector has the potential to promote national economic growth and provide employment opportunities for Nigerians.^10^ For instance, poultry farming serves as an additional occupation to supplement the income of small farm families.^10^ Similarly, the poultry value food chain accounts for about a quarter of local meat production in Nigeria and promotes the per capita animal protein consumption of the population.^11^ Despite these advantages, small-scale poultry farmers in Nigeria lose up to 18% of chicks in the first two weeks of rearing, often due to salmonellosis; this negatively impacts food security and the socio-economic status of farmers.^12^

The expansion of the poultry industry has increased the occurrence of salmonellosis, which has become an important public health threat in Nigeria, causing heavy economic loss and higher human and animal morbidities and mortalities.^13,14^ The most prevalent human *Salmonella* serovars are also common in poultry, suggesting a possible epidemiologic interface between human infections and poultry as a reservoir of *Salmonella.*^8^ *Salmonella* can contaminate poultry and propagate among breeding flocks and at all stages of the production cycle, including during hatching, transportation, slaughtering, processing and retailing, and subsequently spread to humans.^15^

Antimicrobial resistance is an emerging global threat associated with the reduced efficacy of antimicrobials in the treatment of human and animal infections, prolonged morbidity, and death.^16^ Multidrug-resistant organisms are an increasing challenge to human and livestock health worldwide, and Nigeria is not excluded from the antimicrobial resistance burden. Antimicrobials are used in veterinary practice as growth promoters, prophylactics or therapeutics. The indiscriminate use of antimicrobials has been linked to increased antimicrobial resistance among bacterial pathogens isolated from poultry and the poultry environment, including *Salmonella*.^17^ Therefore, poultry may harbour multidrug-resistant pathogens, which could be transmitted to human populations anywhere through direct contact or by consumption. Hence, our study focused on determining the occurrence and antimicrobial susceptibility patterns of *Salmonella* isolates from chickens in poultry farms in Ibadan, Oyo State, Nigeria.

## Methods

### Ethical considerations

The Animal Care and Use Research Ethics Committee (ACUREC), University of Ibadan, Nigeria, reviewed and approved the protocols of the study (UI-ACUREC/18/0105). Verbal informed consent was obtained from farm owners before sampling chickens.

### Study design and sampling method

A cross-sectional study was conducted to determine the occurrence of multidrug-resistant *Salmonella* in poultry farms in Ibadan, Oyo State, Southwest Nigeria. A multistage sampling technique was used to select the chickens for sampling. First, five local government areas (LGAs) in Ibadan metropolis, namely Ibadan North, Ibadan North East, Ibadan North West, Ibadan South West and Ibadan South East were randomly selected for the study. Next, a total of 10 commercial layer poultry farms (A–J), two from each of the five LGAs, were purposively recruited based on the presence of commercial layer production and farmers’ consent. Afterwards, a single pen from each farm was randomly selected, from which 36 chickens were sampled by systematic random sampling. The population of the poultry farms ranged from 500 to 2000 chickens aged between 15 and 36 weeks at the time of sampling; the breeds raised were Isa Brown and Bovans Nera Black. The farms all operated an intensive management system using battery cages.

### Sample size

Using a prevalence of 3.5% of *Salmonella* species reported in layers from poultry farms in Ilorin, Kwara State,^18^ the sample size was calculated using the formula,^19^

$$n=z^{2}p(1-p)/d^{2}$$ [Eqn 1]

where *n* = minimum sample number, *z* = confidence interval at 95% (1.96), *p* = prevalence level of subject (3.5%), and *d* = level of precision (2%). A sample size of 324 was determined, and a 10% non-contingency was added to make up for non-response, giving a minimum target sample size of 360.

### Sample collection

All sanitary and biosecurity protocols were observed by investigators to avoid the spread of pathogens between farms during sample collection. Farm visits were conducted at about 2-week intervals over eight months from 4 April 2018 to 20 November 2018. Each farm was visited once by the investigators and 36 samples were collected during each visit. The investigators visited each farm on dates specified by the farm managers to ensure that no biosecurity measures were compromised. A systematic random sampling was performed to collect samples from chickens within the pens irrespective of age, breed, and health status. A total of 360 cloacal swabs were collected from live chickens (layers) for the detection of *Salmonella*. Samples from farms A and C were taken from diseased chickens as the farmers refused access to the apparently healthy ones.

Cloacal swab samples were collected by gently rotating the cotton swab within the cloacae, with care taken to avoid contact with other areas to prevent contamination. All samples were transported in sterile 10 mL tubes on ice at 4 °C to the Veterinary Public Health Laboratory of the Department of Veterinary Public Health and Preventive Medicine, University of Ibadan, for bacteriological analysis. No transport medium was used. Samples were processed immediately on arrival at the laboratory. For each sampled chicken, information on the species, breed, age, health status, management system, and recent antibiotics administered was also obtained and documented.

### Isolation of *Salmonella*

Isolation of *Salmonella* was carried out according to the International Organization for Standardization standard 6579 guidelines^20^ for isolation and characterisation of *Salmonella* species. Briefly, pre-enrichment was conducted by transferring each swab into 10 mL tubes containing buffered peptone water (Oxoid, Basingstoke, United Kingdom). Each tube was then thoroughly homogenised by vortexing and incubated at 37 °C for 18–20 h. Subsequently, 0.1 mL of the pre-enrichment broth culture was inoculated into 10 mL of Rappaport Vassiliadis broth (Oxoid, Basingstoke, United Kingdom) for the selective enrichment of *Salmonella* and incubated at 42 °C for 24 h. A loopful from the overnight enrichment broth was then inoculated onto xylose lysine deoxycholate agar (Oxoid, Basingstoke, United Kingdom) and brilliant green agar (Oxoid, Basingstoke, United Kingdom), and the plates were incubated at 37 °C for 24 h. To obtain pure colonies, the presumptive *Salmonella* colonies that appeared red with black centres on xylose lysine deoxycholate agar and pinkish-white or red surrounded by a red halo on brilliant green agar were subcultured on xylose lysine deoxycholate agar plates and incubated at 37 °C for 24 h. The pure cultures were subsequently inoculated on nutrient agar slants, incubated at 37 °C for 24 h and stored in the fridge at 4 °C.

### Phenotypic characterisation of *Salmonella*

*Salmonella* isolates were identified based on colony morphology, and further phenotypically confirmed and characterised using Gram staining (negative) and standard laboratory biochemical tests such as indole (negative), methyl red (positive), Voges-Proskeur (negative), citrate (negative), oxidase (negative), catalase (positive), urease (negative), and triple sugar iron agar (acid/alkaline). Also, sugar fermentation tests for glucose (positive), lactose (negative), mannitol (positive), sucrose (negative), and xylose (positive) were performed.

### Antimicrobial susceptibility tests

The antimicrobial susceptibility test was carried out using the agar disk diffusion method.^21^ The sensitivity patterns of the cultured isolates were reported for standard antibiotics according to the Clinical Laboratory Standards Institute.^22^ Briefly, putative *Salmonella* isolates on nutrient agar slants were subcultured onto nutrient agar plates and incubated for 24 h at 37 °C. Subsequently, a colony from each plate was emulsified into sterile saline to achieve turbidity equivalent to 0.5 McFarland standard (~ 10^8^ CFU/mL). The inoculum was aseptically and uniformly spread on the surface of prepared Muller Hinton agar plates (Oxoid, Basingstoke, United Kingdom). Thereafter, the antibiotics disks (Biomark Laboratories, Pune, Maharashtra, India) were placed aseptically on the inoculated agar surface using sterile forceps and the plates were incubated at 37 °C for 16–18 h. The following antimicrobials were used: tetracycline (10 µg), cotrimoxazole (25 µg), gentamicin (10 µg), cefuroxime (30 µg), chloramphenicol (10 µg), ciprofloxacin (5 µg), meropenem (10 µg) and amikacin (30 µg). The zones of inhibition around each disk were measured and interpreted according to the Clinical Laboratory Standards Institute guidelines. The *Escherichia coli* American Type Culture Collection 25922 reference strain was used as the control. An isolate was considered multidrug-resistant if resistant to at least one antimicrobial in three or more antimicrobial classes.

### Data analysis

Data were analysed using descriptive statistics such as proportions and percentages and presented as a table and figures. A Pearson’s chi-squared (χ^2^) test and odds ratios were computed to test the association between the presence of *Salmonella* and age, breed, farm location, and health status of the chickens. The level of significance was set at *p ≤* 0.05. All statistical analyses were conducted using the Statistical Package for the Social Sciences version 21 (IBM Corp., Armonk, New York, United States, 2012).

## Results

**FIGURE 1:**
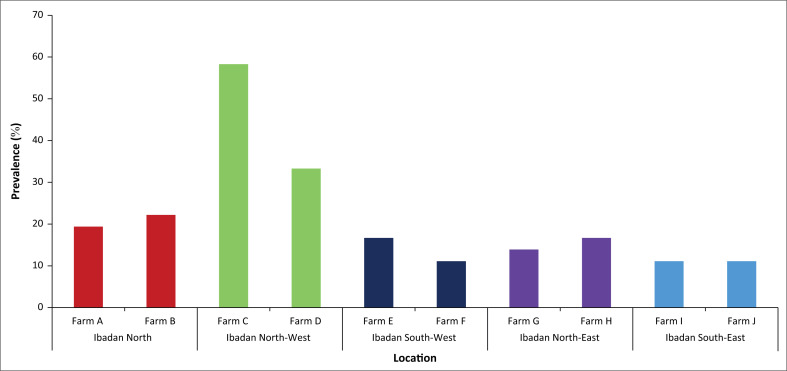
Occurrence of *Salmonella* species in chickens from poultry farms in Ibadan metropolis, Nigeria, April 2018 – November 2018.

A total of 77 (21.4%) of the 360 samples were positive for *Salmonella.* Farm C, one of the farms in Ibadan North-West where only sick chickens were sampled, had the highest *Salmonella* prevalence (21/36; 58.3%) (Figure 1). Overall, the prevalence of *Salmonella* was higher among chickens of the Isa Brown breed (51/216; 23.6%) and chickens aged between 21 and 26 weeks (39/108; 36.1%) (Table 1).

**TABLE 1:** Association between *Salmonella* prevalence and demographic variables of chickens from poultry farms in Ibadan metropolis, Nigeria, April 2018 – November 2018.

Characteristic	Variables	*Salmonella* detected		*Salmonella* not detected		OR	95% CI	*p*
		*N*	%	*N*	%			
**Breed**	Isa Brown (*n* = 216)	51	23.6	165	76.4	1.40	0.83–2.38	0.26
	Bovans Nera Black† (*n* = 144)	26	18.1	118	81.9	Ref	-	-
	Total (*n* = 360)	77	-	283	-	-	-	-
**Age distribution**	15–20 weeks† (*n* = 72)	8	11.1	64	88.9	Ref.	-	-
	21–26 weeks (*n* = 108)	39	36.1	69	63.9	4.52	1.97–10.40	< 0.001*
	27–32 weeks (*n* = 72)	11	15.3	61	84.7	1.44	0.54–3.83	0.62
	33–38 weeks (*n* = 108)	19	17.6	89	82.4	1.71	0.70–4.14	0.33
	Total (*n* = 360)	77	-	283	-	-		-
**Health status**	Sick (*n* = 72)	28	38.9	44	61.1	0.32	0.18–0.57	< 0.001*
	Healthy† (*n* = 288)	49	17.0	239	83.0	Ref.	-	-
	Total (*n* = 360)	77	-	283	-	-	-	-
**Location**	Ibadan North† (*n* = 72)	15	20.8	57	79.2	Ref.	-	-
	Ibadan North-West (*n* = 72)	33	45.8	39	54.2	3.22	1.54–6.70	0.003*
	Ibadan South-West (*n* = 72)	10	13.9	62	86.1	0.61	0.26–1.47	0.38
	Ibadan North-East (*n* = 72)	11	15.3	61	84.7	0.69	0.29–1.62	0.52
	Ibadan South-East (*n* = 72)	8	11.1	64	88.9	0.48	0.19–1.20	0.17
	Total (*n* = 360)	77	-	283	-	-	-	-

OR, odds ratio; CI, confidence interval.

* , *P*-values ≤ 0.05 are statistically significant.

† , Reference group.

Age, farm location, and health status of chickens were all associated with *Salmonella* carriage. Layer chickens aged between 21 and 26 weeks had four times the odds of *Salmonella* carriage compared to those aged between 15 and 20 weeks (odds ratio = 4.52; 95% confidence interval: 1.97–10.4, *p* < 0.001). Similarly, chickens in farms in Ibadan North-West had three times higher odds of being *Salmonella*-positive compared to those from Ibadan North LGA (odds ratio = 3.22; 95% confidence interval: 1.54–6.7; *p* = 0.003). Farms in Ibadan North-West LGA operated under poor hygienic and low biosecurity conditions. Interestingly, sick chickens had lower odds of harbouring *Salmonella* than the healthy ones (odds ratio = 0.32; 95% confidence interval: 0.18–0.57; *p* < 0.001).

### Antimicrobial resistance profiles of the *Salmonella* isolates

**FIGURE 2:**
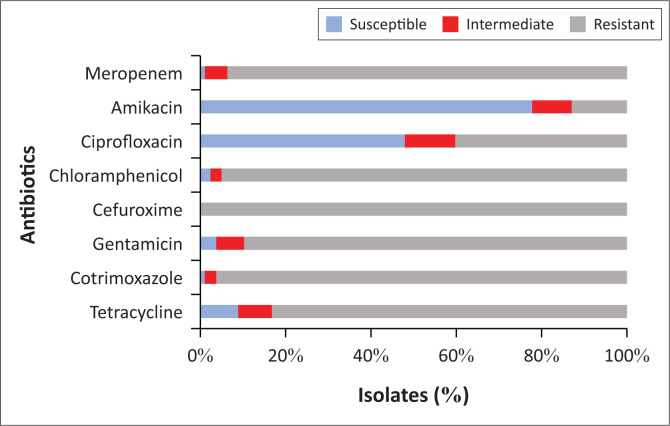
Antimicrobial resistance profile of *Salmonella* isolates from chickens from poultry farms in Ibadan metropolis, Nigeria, April 2018 – November 2018.

All of the isolates (*n* = 77; 100.0%) were resistant to cefuroxime. Isolates were also highly resistant to cotrimoxazole (*n* = 74; 96.1%), chloramphenicol (*n* = 73; 94.8%), meropenem (*n* = 72; 93.5%), gentamicin (*n* = 69; 89.6%) and tetracycline (*n* = 64; 83.1%), but only moderately resistant to ciprofloxacin (*n* = 31; 40.3%) (Figure 2).

## Discussion

Food safety issues, including the presence of zoonotic and antibiotic-resistant pathogens in farm animals, are of increasing concern among consumers of animal food products globally. We reported a 21.4% prevalence of *Salmonella* in commercial poultry in Ibadan. Previous studies have reported the prevalence of *Salmonella* infection in poultry farms and chickens in Nigeria to be between 2.0% and 43.0%,^17,23,24,25,26,27,28,29,30,31,32^ and this is similar to the prevalence reported in another study conducted in Bangladesh.^33^ *Salmonella* have been implicated in bacteraemia, septicaemia and gastroenteriti*s* in humans, and their presence in poultry chickens may pose serious health risks to humans.^34^ The observed *Salmonella* prevalence in the farms studied may be due to poor biosecurity measures, contamination from poultry premises or feed, or bacterial transmission via faecal matter by insects and rodents.^35^ Chickens from poultry farms in Ibadan North-West LGA had three times the odds of being colonised by *Salmonella* and this may be attributed to the unhygienic and poor sanitary measures observed on the farms during site visits, including the presence of heaps of faecal matter in the pens and absence of disinfectant foot dips. *Salmonella* transmission in farms may also be promoted by inadequate knowledge and poor attitudes towards biosecurity and poultry management.^36^ Similarly, as observed, leaving dead carcasses in the farm, lack of proper hygiene, and the presence of faeces in water could have accounted for the high prevalence of *Salmonella* in farms in Ibadan North-West. Similar to past studies conducted in Nigeria, Colombia and Baghdad,^37,38,39^ there was no association between chicken breed and *Salmonella* colonisation in our study.

The observed high rates of resistance to antimicrobials among the isolates is not surprising as most poultry farmers in the country have unlimited over-the-counter access to antimicrobials and indiscriminately use them as growth promoters and prophylactics^40^ without veterinary prescriptions.^41,42^ The ciprofloxacin resistance was lower in this study compared to another study conducted in Nigeria,^43^ but similar to a report from the United States.^44^

All *Salmonella* isolates were resistant to cefuroxime, which was the only cephalosporin tested in this study; this is similar to previous reports from Nigeria, the United Arab Emirates, Germany and India.^17,45,46^ This poses a serious threat to humans as cephalosporin-resistant *Salmonella* can potentially be transmitted to humans via the consumption of such poultry or poultry products. Moreover, there are limited treatment options for human infections caused by pathogens resistant to multiple classes of antimicrobials, particularly those involving the cephalosporins and fluoroquinolones.^46^

The high resistance to tetracycline documented in this study is not surprising. Tetracycline is the most abused antibiotic for the treatment and prevention of poultry disease in Nigeria.^17,40,41^ Nevertheless, the tetracycline resistance rate in this study is higher than those reported in past studies in Ghana^47^ and Cote d’Ivoire,^48^ possibly because of differences in geographical location, farm biosecurity levels, and national implementation of antimicrobial policies in these countries.

### Limitations

We were not able to identify the specific serovars of the *Salmonella* isolates due to non-availability of antisera to perform serotyping. Also, the findings of this study are not generalisable to farms in Ibadan as only the five LGAs in Ibadan metropolis were sampled. Further work, which should involve the detailed sampling of more LGAs and serotyping of *Salmonella* isolates, is needed to provide more insight into the prevalence of the various serovars circulating in the poultry population in Ibadan, Nigeria.

### Conclusion

We report a high prevalence of *Salmonella* among chickens from poultry farms in Ibadan, Nigeria. Apparently healthy and sick chickens may serve as potential shedders and propagators of drug-resistant *Salmonella*, with possible implications for consumer health and the environment. Data generated from this study could inform policies or guidelines for the improvement of on-farm hygiene and biosecurity best practices among poultry farmers to reduce the burden of *Salmonella* in the industry. Also, Nigeria urgently needs well-coordinated or integrated national surveillance systems and networks to monitor antimicrobial use and resistance in animal and human populations and the environment.

## References

[1] Thames HT, Theradiyil Sukumaran A. A review of Salmonella and campylobacter in broiler meat: Emerging challenges and food safety measures. Foods. 2020;9(6):776–798. 10.3390/foods9060776 PMC7353592 32545362

[2] Bula-Rudas FJ, Rathore MH, Maraqa NF. Salmonella infections in childhood. Adv Pediatr. 2015;62(1):29–58. 10.1016/j.yapd.2015.04.005 26205108

[3] John A, Crump JA, Sjölund-Karlsson M, Gordon MA, Parry CM. Epidemiology, clinical presentation, laboratory diagnosis, antimicrobial resistance, and antimicrobial management of invasive Salmonella infections. Clin Microbiol Rev. 2015;28(4):901–937. 10.1128/CMR.00002-15 26180063 PMC4503790

[4] Abebe E, Gugsa G, Ahmed M. Review on major food-borne zoonotic bacterial pathogens. J Trop Med. 2020;13:4674235. 10.1155/2020/4674235 PMC7341400 32684938

[5] Jajere SM. A review of Salmonella enterica with particular focus on the pathogenicity and virulence factors, host specificity and antimicrobial resistance including multidrug resistance. Vet World. 2019;12(4):504–521. 10.14202/vetworld.2019.504-521 31190705 PMC6515828

[6] Smith SI, Seriki A, Ajayi A. Typhoidal and non-typhoidal Salmonella infections in Africa. Eur J Clin Microbiol Infect Dis. 2016;35:1913–1922. 10.1007/s10096-016-2760-3 27562406

[7] Feasey NA, Dougan G, Kingsley RA, Heyderman RS, Gordon MA. Invasive non-typhoidal Salmonella disease: An emerging and neglected tropical disease in Africa. Lancet. 2012;379(9835):2489–2499. 10.1016/S0140-6736(11)61752-2 22587967 PMC3402672

[8] Abdullahi B, Abdulfatai K, Wartu JR, Mzungu I, Muhammad HID, Abdulsalam AO. Antibiotics susceptibility patterns and characterization of clinical Salmonella serotypes in Katsina State, Nigeria. Afr J Microbiol Res. 2014;8(9):915–921. 10.5897/AJMR12.2253

[9] Dagnew B, Haile A, Girmay M, Tadesse E. Prevalence and antimicrobial susceptibility of Salmonella in poultry farms and in-contact humans in Adama and Modjo towns, Ethiopia. Microbiology Open. 2020;9(8):e1067. 10.1002/mbo3.1067 32510864 PMC7424249

[10] Odine AI, Ibrahim FD, Usman RK, Jirgi AJ, Ibrin S, Audu JF. Economic efficiency of poultry egg production in Kogi State, Nigeria. J Biol Agric Healthcare. 2015;5(14):180–186.

[11] Adebayo OO, Adeola RG. Socioeconomic factors affecting poultry farmers in local area of Osun State. Hum Ecol J. 2005;18(1):39–41.

[12] Agbaje M, Davies R, Oyekunle MA, Ojo OE, Fasina FO, Akinduti PA. Observation on the occurrence and transmission of Salmonella gallinarum in commercial poultry farms in Ogun State, South Western Nigeria. Afr J Microbiol Res. 2010;4(9):796–800.

[13] Fagbamila IO, Barco L, Mancin M, et al. Salmonella serovars and their distribution in Nigerian commercial chicken layer farms. PLoS One. 2017;12(3):e0173097. 10.1371/journal.pone.0173097 28278292 PMC5344354

[14] Muhammed M, Muhammed LU, Ambali AG, Mani AU, Azard S, Barco L. Prevalence of Salmonella associated with chick mortality at hatching and their susceptibility to antimicrobial agents. Vet Microbiol. 2010;140(1–2):131–135. 10.1016/j.vetmic.2009.07.009 19643554

[15] Jibril AH, Okeke IN, Dalsgaard A, et al. Prevalence and risk factors of Salmonella in commercial poultry farms in Nigeria. PLoS One. 2020;15(9):e0238190. 10.1371/journal.pone.0238190 32966297 PMC7510976

[16] Foley SL, Lynne AM, Nayak R. Salmonella challenges: Prevalence in swine and poultry and potential pathogenicity of such isolates. J. Anim Sci. 2008;86:149–162. 10.2527/jas.2007-0464 17911227

[17] Liselotte Diaz Högberg LD, Heddini A, Cars O. The global need for effective antibiotics: Challenges and recent advances. Trends Pharmacol Sci. 2010;31(11):509–515. 10.1016/j.tips.2010.08.002 20843562

[18] Agada GOA, Abdullahi IO, Aminu M, et al. Prevalence and antibiotic resistance profile of Salmonella isolates from commercial poultry and poultry farm-handlers in Jos, Plateau State, Nigeria. Br Microbiol Res J. 2014;4(4):462–479. 10.9734/BMRJ/2014/5872

[19] Thrusfield M. Veterinary epidemiology. 3rd ed. Oxford: Blackwell Science Ltd; 2007.

[20] International Standard Organization. ISO 6579-1:2017: Microbiology of the food chain – Horizontal method for the detection, enumeration and serotyping of *Salmonella* – Part 1: Detection of Salmonella spp. 1st ed.; 2017: p. 1–50. Geneva, Switzerland.

[21] Bauer AW, Kirby WM, Sherris JC, Turck M. Antibiotic susceptibility testing by a standardized single disk method. Am J Clin Pathol. 1966;45(4):493–496. 10.1093/ajcp/45.4_ts.493 5325707

[22] Clinical and Laboratory Standards Institute. CLSI Guidelines. Performance standards for antimicrobial susceptibility testing. 28th ed. 2018. Wayne, PA.

[23] Adesiji YO, Alli OT, Adekanle MA, Jolayemi JB. Prevalence of Arcobacter, Escherichia coli, Staphylococcus aureus and Salmonella species in retail raw chicken, pork, beef and goat meat in Osogbo, Nigeria. J Biomed Res. 2011;3(1):8–12. 10.4314/sljbr.v3i1.66644

[24] Ukut IO, Okonko IO, Ikpoh IS, et al. Assessment of bacteriological quality of fresh meats sold in Calabar metropolis, Nigeria. Electron J Environ Agr Food Chem. 2010;9(1):89–100.

[25] Saba AB, Olatoye IO, Oridupa OA, Odiaka C. Antibiotic resistance in *Salmonella* sp. and *Escherichia coli* isolated from poultry in Ibadan, Nigeria. Trop Vet. 2012;30(2):55–64.

[26] Adeyanju GT, Ishola O. Salmonella and Escherichia coli contamination of poultry meat from a processing plant and retail markets in Ibadan, Oyo state, Nigeria. Springerplus. 2014;3:139–148. 10.1186/2193-1801-3-139 25674440 PMC4320193

[27] Olatoye IO. Antibiotics use and resistance patterns of Salmonella species in poultry from Ibadan, Nigeria. Trop Vet. 2011;29(2):28–35.

[28] Babatunde SK, Kolawole DO, Adedayo MR, Ajiboye AE, Ajao AT, Mustapha ON. Prevalence and characterization of Salmonella isolates from poultry farms in Ilorin, Nigeria. J Life Sci Res. 2017;4(1):1–4. 10.20448/journal.504.2017.41.1.4

[29] Uhunmwangho EJ, Okhia O, Eruotor OH, Blackies HOT, Uhunmwangho A. The prevalence of *Salmonella* species among poultry chickens in Ekpoma Edo-state, Nigeria. Int J Community Res. 2014;3(4):101–105.

[30] Fashae K, Ogunsola F, Aarestrup FM, Hendriksen RS. Antimicrobial susceptibility and serovars of *Salmonella* from chickens and humans in Ibadan, Nigeria. J Infect Dev Ctries. 2010;4(8):484–494. 10.3855/jidc.909 20818100

[31] Mbuko IJ, Raj MA, Ameh J, Saidu L, Musa WI, Abdul PA. Prevalence and seasonality of fowl typhoid disease in Zaria-Kaduna State, Nigeria. J Bacteriol Res. 2009;1(1):001–005.

[32] Aje Anejo‑Okopi J, Ejiji Isa S, Audu O, Fagbamila IO, Iornenge JC, Smith IS. Isolation and polymerase chain reaction detection of virulence invA gene in Salmonella spp. from poultry farms in Jos, Nigeria. J Med Tropics. 2016;18(2):98–102. 10.4103/2276-7096.192237

[33] Islam M, Hassan J, Khan MR. Seroprevalence of Mycoplasma gallisepticum infection in backyard and commercial layer chickens in Bhola district, Bangladesh. J Adv Vet Anim Res. 2014;1(1):11–15. 10.5455/javar.v1i1p11-15

[34] Manoj J, Singh MK, Singh YP. The role of poultry in food borne salmonellosis and its public health importance. Adv Anim Vet Sci. 2015;3(9):485–490. 10.14737/journal.aavs/2015/3.9.485.490

[35] Jones FT. A review of practical Salmonella control measures in animal feed. J Appl Poult Res. 2011;20(1):102–113. 10.3382/japr.2010-00281

[36] Raufu IA, Ahmed OA, Aremu A, Odetokun IA, Raji MA. Salmonella transmission in poultry farms: The roles of rodents, lizards and formites. Savannah Vet J. 2019;2:1–4.

[37] Ibrahim T, Yakubu N, Grace P, et al. Antimicrobial resistance profile of Salmonella Typhimurium isolated from commercial poultry and poultry farm handlers in Nasarawa State, Nigeria. Microbiol Res J Int. 2019;28(4):1–12. 10.9734/mrji/2019/v28i430136

[38] Rodriguez JM, Rondón IS, Verjan N. Serotypes of Salmonella in broiler carcasses marketed at Ibague, Colombia. Braz J Poultry Sci. 2015;17(4):545–552. 10.1590/1516-635X1704545-552

[39] Al-Khayat L, Khammas EJ, Ali RM, Al-Owaini BA, Abdulrasool LMS. Rapid detection and identification of poultry *Salmonella* serotypes using multiplex PCR assay. MRVSA. 2016;5(3):31–40. 10.22428/MRVSA.5.3-5.03102016

[40] Adebowale OO, Adeyemo OK, Awoyomi O, Dada R, Adebowale O. Antibiotic use and practices in commercial poultry laying hens in Ogun state Nigeria. Rev Elev Med Vet Pays Trop. 2016;69(1):41–45. 10.19182/remvt.31170

[41] Awogbemi J, Adeyeye M, Akinkunmi EO. A survey of antimicrobial agents usage in poultry farms and antibiotic resistance in Escherichia coli and Staphylococci isolates from the poultry in Ile-Ife, Nigeria. J Infect Dis Epidemiol. 2018;3:47–55.

[42] Adebowale OO, Adeyemo FA, Bankole N, et al. Farmers’ perceptions and drivers of antimicrobial use and abuse in commercial pig production, Ogun State, Nigeria. Int J Environ Res Public Health. 2020;17(10):3579–3599. 10.3390/ijerph17103579 PMC7277550 32443741

[43] Salihu AE, Onwuliri FC, Mawak JO. Antimicrobial resistance profiles of Salmonella gallinarum isolates from free-range chickens in Nasarawa state, Nigeria. Int J Bacteriol Res. 2014;2(1):19–27.

[44] Malorny B, Hoofa J, Bunge C, Helmuth R. Multicenter validation of the analytical accuracy of Salmonella PCR: Towards an international standard. Appl Environ Microbiol. 2013;69(1):290–296. 10.3390/ijerph17103579 PMC152403 12514007

[45] Rotimi VO, Jamal W, Pal T, Sonnevend A, Dimitrov TS, Albert MJ. Emergence of multidrug-resistant Salmonella spp. and isolates with reduced susceptibility to ciprofloxacin in Kuwait and the United Arab Emirates. Diagn Microbiol Infect Dis. 2008;60(1):71–77. 10.1016/j.diagmicrobio.2007.07.007 17931817

[46] Sivakumar T, Avinash Saravanavel N, Prabhu D, Shankar T, Vijayabaskar P. Characterization of multidrug resistant patterns of Salmonella sp. World J Med Sci. 2012;7(2):64–67.

[47] Andoh LA, Dalsgaard A, Obiri-Danso K, Newman MJ, Barco L, Olsen JE. Prevalence and antimicrobial resistance of Salmonella serovars isolated from poultry in Ghana. Epidemiol Infect. 2016;144(15):3288–3299. 10.1017/S0950268816001126 27334298 PMC9150280

[48] Bonny AC, Karou ATG, Sanogo M, Atobla K, Ahonzo-Niamke SL. Prevalence of Salmonella and their antibiotic susceptibility patterns in the District of Abidjan, Côte d’Ivoire. Int J Biol Chem Sci. 2014;8(2):450–458. 10.4314/ijbcs.v8i2.5

